# Climatic Niche Conservatism and Biogeographical Non-Equilibrium in *Eschscholzia californica* (Papaveraceae), an Invasive Plant in the Chilean Mediterranean Region

**DOI:** 10.1371/journal.pone.0105025

**Published:** 2014-08-19

**Authors:** Francisco T. Peña-Gómez, Pablo C. Guerrero, Gustavo Bizama, Milén Duarte, Ramiro O. Bustamante

**Affiliations:** 1 Instituto de Ecología y Biodiversidad, Facultad de Ciencias, Universidad de Chile, Santiago, Chile; 2 Departamento Cs. Ecológicas, Facultad de Ciencias, Universidad de Chile, Santiago, Chile; 3 Departamento de Botánica, Facultad de Ciencias Naturales y Oceanográficas, Universidad de Concepción, Concepción, Chile; Helmholtz Centre for Environmental Research – UFZ, Germany

## Abstract

Species climate requirements are useful for predicting their geographic distribution. It is often assumed that the niche requirements for invasive plants are conserved during invasion, especially when the invaded regions share similar climate conditions. California and central Chile have a remarkable degree of convergence in their vegetation structure, and a similar Mediterranean climate. Such similarities make these geographic areas an interesting natural experiment for testing climatic niche dynamics and the equilibrium of invasive species in a new environment. We tested to see if the climatic niche of *Eschscholzia californica* is conserved in the invaded range (central Chile), and we assessed whether the invasion process has reached a biogeographical equilibrium, i.e., occupy all the suitable geographic locations that have suitable conditions under native niche requirements. We compared the climatic niche in the native and invaded ranges as well as the projected potential geographic distribution in the invaded range. In order to compare climatic niches, we conducted a Principal Component Analysis (PCA) and Species Distribution Models (SDMs), to estimate *E. californica*'s potential geographic distribution. We also used SDMs to predict altitudinal distribution limits in central Chile. Our results indicated that the climatic niche occupied by *E. californica* in the invaded range is firmly conserved, occupying a subset of the native climatic niche but leaving a substantial fraction of it unfilled. Comparisons of projected SDMs for central Chile indicate a similarity, yet the projection from native range predicted a larger geographic distribution in central Chile compared to the prediction of the model constructed for central Chile. The projected niche occupancy profile from California predicted a higher mean elevation than that projected from central Chile. We concluded that the invasion process of *E. californica* in central Chile is consistent with climatic niche conservatism but there is potential for further expansion in Chile.

## Introduction

Understanding the factors that lead to the successful spread of species outside their native ranges is a fundamental issue in the study of biological invasions [1], [2]. The geographic distribution of invasive species is controlled by factors such as climatic requirements, dispersal ability and biotic interactions [3]. Among these factors, species climate requirements are important and can be useful for predicting the geographic spread of species at large spatial scales [4], [5].

Predicting the extent of geographic invasions is based on suppositions stemming from climatic niche conservatism [6]. If the climatic niche is conserved, then the projection of the potential species distribution from native to invaded range is concordant with the projection from the invaded range. These comparative studies are generally done between climatic analogue regions [7].

However, this protocol is controversial because it excludes the possibility to detect niche shifts to non-analogous environments that could be occupied by invasive species in the invaded range. For example, invasive species could occupy new environments (nonanalogous) due to adaptive evolution during invasions, the absence of biotic constraints, the absence of dispersal limitations [8], or because of the availability of suitable conditions present in the invaded range that are not available in their native range [7]. There is evidence of niche conservatism in invasive plants [7]; however there are cases where invasive plants display climatic niche shifts, thus occupying areas within climates that were not expected, based on native climatic requirements [9]–[12].

One assumption commonly used in biogeographical studies is that species are capable of covering the entirety of a given geographic extent that best suits their climatic requirements, rapidly reaching the geographic equilibrium [13]. In the case of invasive species, they may be far from full equilibrium [14] due to of several ecological aspects preventing their establishment and colonization, such as dispersal limitation [15], [16], negative interactions [17], key mutualists may be absent [18], or because these species need time to occupy every suitable location [14], [19].

California and central Chile are two distant geographic areas with very similar Mediterranean climates, topography and vegetation types [20], [21]. These features have stimulated comparative studies which have demonstrated that, despite their similarities, each geographic area has distinctive native and exotic flora [22], [23] differing fire regimes [21], [24], different soil nutrient content [25], and different anthropogenic and economic activities [23]. Overall, these quite distinctive floras have undergone convergent evolution, expressed in similar native vegetation structure and the functional traits of native plants [24], [26], [27].

*Eschscholzia californica* Cham. (Papaveraceae) (California poppy) is a native herb from the west coast of North America, mainly found in California. This species is distributed between latitude 32°and 41° N, in climatic conditions very similar to those recorded in its distribution in Chile [28]. This species is usually found at low elevations (below 2000 m altitude) and thrives in warm temperatures [28]. Naturalized populations are found in Australia, Chile, New Zealand and South Africa, among other countries. Beetles pollinate *E. californica* in its native range and. If pollinated, the resulting fruit is a long, slender pod that dries and splits, shooting tiny round black seeds in all directions. This species persists in the seed bank during dry season, lying dormant as seed for years in some areas. When enough rain falls seeds rapidly germinate and become reproductive in a matter of a few months [28].

*E. californica* was first introduced into Chile in the mid-1800s, *via* botanical gardens [29]. In 1890, the first specimen from a naturalized population was collected near Valparaíso (coastal central Chile). This species is currently distributed in Chile between latitudes 30° and 38° S, and between 0 and 2,200 m altitude [30], [31]. *E. californica* is particularly interesting for studying the niche dynamics because plant size, fecundity, and resistance to herbivores are significantly greater in the invaded range (central Chile) than in the native one [32]–[34].

In this study, we addressed two questions: (i) has *E. californica* conserved its climatic niche in Chile? and (ii) has *E. californica* reached a geographical equilibrium or can we expect it to spread further in Chile? To answer these questions, we carried out climatic niche analyses based on principal component analysis (PCA) [35] to identify parts of the climatic niche spaces that are stable in the invaded range (conserved), and either unfilled or expanded (non-equilibrium cases). We also constructed species distribution models (SDMs) to examine whether this species has achieved geographical equilibrium in central Chile. This information could also be used to plan management practices in order to prevent invasive species from spreading or to contain them.

## Methods

### Occurrence data

Data for the presence of *E. californica* in its native range (California, USA) was compiled from the Consortium of California Herbaria (available at http://ucjeps.berkeley.edu/consortium/, public repository online) and Calflora online databases (available at http://www.calflora.org/, public repository online). The data were carefully filtered according to the following criteria: (i) the data contained associated geo-referenced information (e.g. datum), (ii) they were recorded after 1950 to minimize erroneous geo-referenced information and (iii) they had an associated voucher or were labelled under the name of the botanist who determined the sample. This last criterium was utilized to control taxonomic problems with this species [36].

After pooling and filtering the data, we obtained a total of 311 records for its presence in California. For central Chile, we found 50 registered occurrences at the Herbarium of the University of Concepción (CONC, http://www2.udec.cl/~herbconc/, public repository), and 10 from (SGO, http://www.mnhn.cl/, public repository). To enlarge the dataset, we conducted field campaigns during 2009 and 2010 (Austral spring–summer), covering Andean and coastal ranges from 30° to 38° lat. S, and arrived at a total of 778 occurrences. The dataset is available in Table S1. Duplicated records were removed from both datasets using ENMtools version 1.3 [37].

### Environmental layers

Bioclimatic variables were obtained from the Worldclim database (http://www.worldclim.org/, public repository online) with a spatial resolution of 30 arc-seconds [38]. This data set included a total of 19 bioclimatic variables, which summarized information on temperature and precipitation. Since variable co-linearity may lead to over-fitting [39], we chose a sub-sample of the bioclimatic variables checked for cross-correlation using the Pearson correlation test; only one variable from highly correlated pairs of variables (r>0.90) was included in the model. This procedure was conducted using ENMtools version 1.3 [37].

Our final selection came down to altitude and 14 climatic variables: annual mean temperature (bio1), mean diurnal range (bio 2), isothermality (bio3), temperature seasonality (bio4), maximum temperature of warmest month (bio5), minimum temperature of coldest month (bio6), temperature annual range (bio7), mean temperature of wettest quarter (bio8), mean temperature of driest quarter (bio9), mean temperature of warmest quarter (bio10), mean temperature of coldest quarter (bio11), annual precipitation (bio12), precipitation seasonality (bio15), and precipitation of warmest quarter (bio18). We used ArcGis 9.3 [40] to process the environmental layers.

### Climatic niche

Principal Component Analysis (PCA) [35] was conducted using information from background zones chosen for two study areas, which also included locations for *E. californica* occurrences. This analysis was used to compare the niche of *E. californica* between the native range (California) and the invaded range (central Chile) using the 14 selected bioclimatic variables.

The background zone for California was a polygon within 125°–115° W and 45°–30° N whilst the background for central Chile was a polygon within 74°–70° W and 29°–38° S. For California, this polygon encompasses the California Floristic Province which includes the semiarid, sub-humid region and a portion of the humid region of the area covered by the Mediterranean climate [41]. For central Chile, the chosen polygon is the climatic analogue zone in California as documented in other studies [27].

Occurrence points were converted into density values using a kernel function to smooth the distribution of the densities [35]. These figures were then gridded within the environmental envelope obtained by the previously chosen background zones (10,000 randomly generated points) in the native and invaded range. The occurrence points, converted in occurrence density were ordered along the PCA axes constructed from the climatic environment within the background zones. Two graphical models were constructed for niche analysis: the first, describing the ecological niche of the species in its native range, and the second, describing the ecological niche of the species in the invaded range.

A comparison between the native and the invaded range was used to assess whether *E. californica's* niche has been conserved in central Chile. For this comparison, niche similarity was measured using Schoener's D overlap index [37], the result falling between 0 (no overlap) and 1 (complete overlap). The D overlap index was tested against chance using re-sampling procedures [7], [35], [37]. Following Petitpierre et al. 2012 [7], three regions of the niche, either in the native and invaded range, were considered representative of niche dynamics for invasive species:

1. Stability niche area (S); the area shared between niches of the native and the invaded range. This area is an estimation of niche conservatism (S is the proportion of the densities in the invaded niche that overlap the native niche) [7];
2. The unfilling niche area (U); this is the niche space of the native range that isn't shared with the niche of the species in the invaded range and this area indicates the potential space of the invaded niche that has not yet been occupied in the native range. In other words, U is an estimation of how far the species is from geographical equilibrium (U is the proportion of densities in the native niche found in different conditions to the invaded niche) [7];
3. The expanded niche zone (E); the niche zone in the invaded range which is not shared with the native niche. This area is the degree of niche shift; it indicates new climatic environments occupied by the species in the invaded range (E is the proportion of densities in the invaded niche found in different conditions to the native niche) [7].

These estimations were made considering 75% climatic similarity between the native and invaded range climatic envelopes (following [7]). All the analyses were performed using R software (version 2.15.1) [42] using the function proposed in [35] and [7].

### Predicted distributions

From climatic variables and occurrence data, we constructed ‘species distributions models’ (SDMs) for *E. californica* in its native and invaded range using Maxent [43], a machine-learning method that assesses the distribution probability of a species by estimating the distribution probability of maximal entropy [43]. This software generally performs better than other software commonly used for SDMs using presence-only datasets [44]–[46].

We divided the presence data into two parts: 75% for training and 25% for testing the model. The model's performance was evaluated using the AUC (area under the ROC curve [43]). AUC is a composite measure for model performance and provides a global comparison of model fitting relative to a random prediction. AUC ranges from 0.5 for a model that performs no better than chance to 1.0 for perfect ability to predict presence [43], [47]. For SDMs, we used the same regional background chosen for the Californian niche analysis and the central Chilean analysis.

For SDMs regularization, we smoothed the models to avoid over-parameterization [43], [48]. The regularization refers to smoothing the model, making it more regular, so as to avoid fitting too complex a model [48]. In Maxent, the fit of the model is measured at the occurrence sites, using a “log likelihood” (see Box 1 in [48]). A highly complex model will have a high log likelihood, but may not generalize well [43], [48]. The aim of regularization is to trade off model fit and model complexity [43], [48]. The smoothing was achieved by modifying a β parameter i.e. the value that smoothens the model, making it more regular. β = 1 was the best choice as it led to the most conservative model [48], penalizing over-parameterization (Table 1).

**Table 1:** Parameters of species distribution models (SDMs) for *Eschscholzia californica* in central Chile projected from the invaded range (central Chile) and the native range (California).

Range	N° occurrences	β	Parameters	Threshold	AUC_train._	AUC_test_
Invaded range	778	1	118	0.13	0.936	0.919
Native range	311	1	24	0.13	0.918	0.881

The threshold values correspond to P(occurrence) observed in the lowest percentile (10%) of the occurrence distribution for each range. The β parameter is a measure of SDM regularization. We used β = 1 because it generates the most conservative models by penalizing over-parameterization.

From each occurrence point in central Chile, we extracted a prediction for occurrence probability from the distribution models. Probability values below the 10% percentile [49] were discarded under the assumption that these figures represented unsuitable climatic zones. In both cases the threshold was 0.13. To compare distribution models, we projected potential distribution from the native range (California) to central Chile, and compared it with the projected potential distribution for central Chile [50].

Both SDMs were replicated 100 times, and then averaged. Finally, to predict altitudinal distribution of *E. californica* in central Chile, we generated prediction for niche occupancy profiles [47] from the native and invaded ranges to the invaded range. This method integrates the occurrence probabilities for SDMs (raw data) over a climatic layer, generating a profile for habitat suitability across environmental gradient [47]; in this study we used altitudinal gradient.

## Results

### Climatic niche

**Figure 1:**
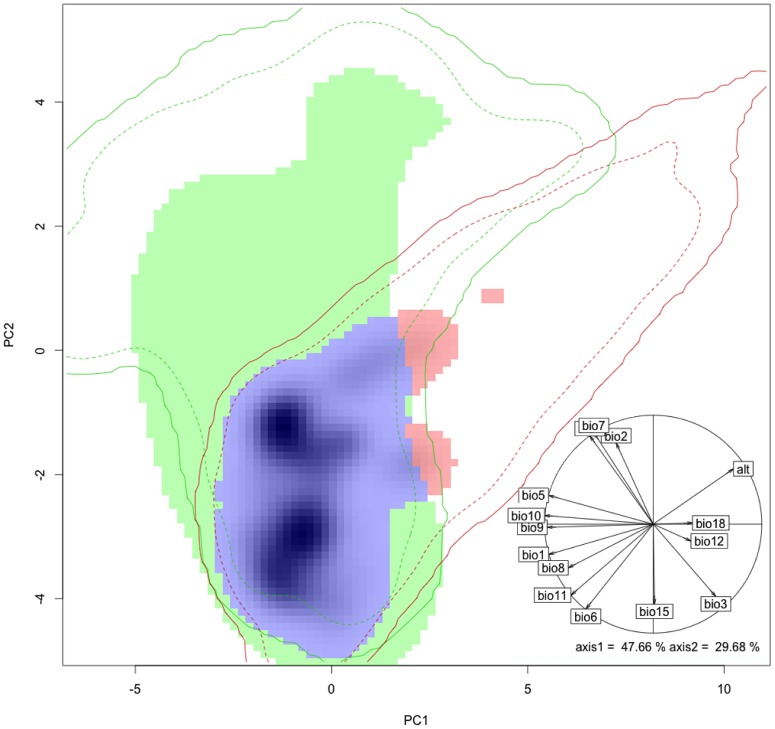
Principal Component Analysis (PCA). The climatic niche of *Eschscholzia californica* in the native range in California (green) and in the invaded range in central Chile (red). The blue area corresponds to niche areas shared in both ranges (niche stability).The solid and dashed lines show 100% and 75% of the climatic envelope from the native (green) and from the invaded range (red), respectively. The green area is the unfilled climatic niche space in the invaded range, and the red area, is the expanded climatic niche in the invaded range. The more intense blue cells represent zones with higher occurrence densities in the invaded niche (central Chile). In the PCA analysis the first axis accounts for 47.66% of the total variance and mainly represents mean annual precipitation, precipitation during the warmest quarter and altitude; the second PCA axis accounts for 29.68% of the total variance and mainly represents precipitation seasonality. In the correlation circle, the hidden label (behind bio7) corresponds to temperature seasonality (bio4).

The niches of California and central Chile were significantly more similar than chance (D = 0.428; Figure 1), from California to central Chile (P = 0.001) and from central Chile to California (P = 0.04). More specifically, the niche in central Chile was almost completely included (nested) within the Californian niche (S = 0.993) (Figure 1). Moreover, the native climatic niche includes a space not shared with the climatic niche in the invaded range (U = 0.532) and the climatic niche of central Chile had a reduced expansion (E = 0.006) (Figure 1).

### Predicted distributions

**Figure 2:**
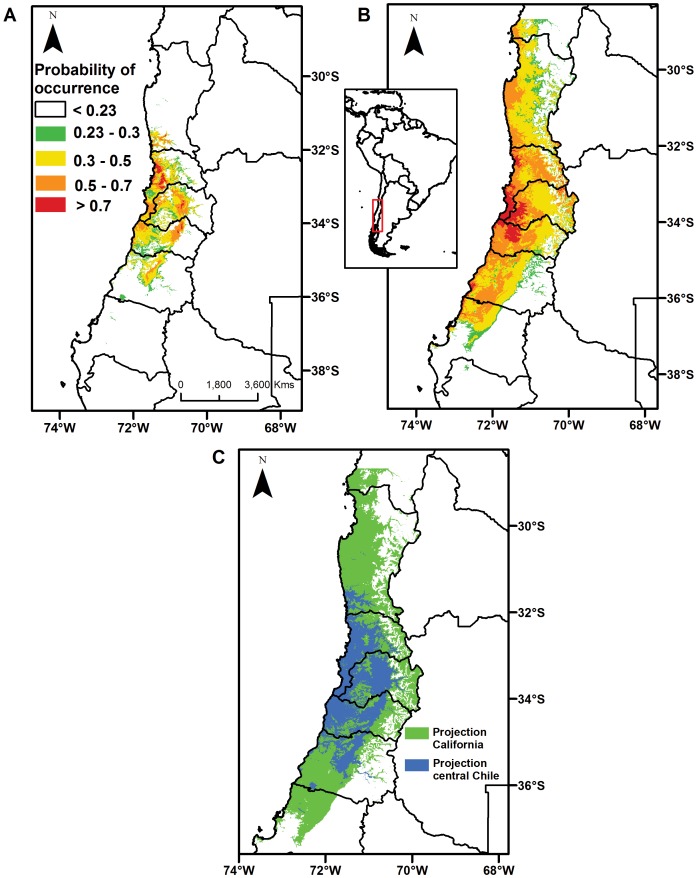
Species distribution models (SDMs) for *Eschscholzia californica* in central Chile. A) Generated with occurrences recorded in the invaded range (central Chile). B) Generated with occurrences recorded in the native range (California). C) Overlap of both SDMs. It shows that 99.9% of the invaded range predicted from central Chile (blue) is included in the area predicted from California; 65% of the area predicted from California (green) is not predicted by the SDM from central Chile; only a small proportion of area (0.1%) in Chile (red) is not predicted from California. AUC values for these models are displayed in Table 1.

The projected distribution models performed well (Table 1). In the invaded range, the predicted distribution of *E. californica* from central Chile covers 48,391 km^2^ (Figure 2A) and the projection from California covers 139,526 km^2^ (Figure 2B). Comparisons between these projected distributions revealed that almost the complete projected distribution from central Chile was included in the projected distribution from California (99.9%). A large portion of the projected distributions (65%) was also predicted only from California model. This area extended approximately 2° further north and approximately 1° further to the south (Figure 2C).

**Figure 3:**
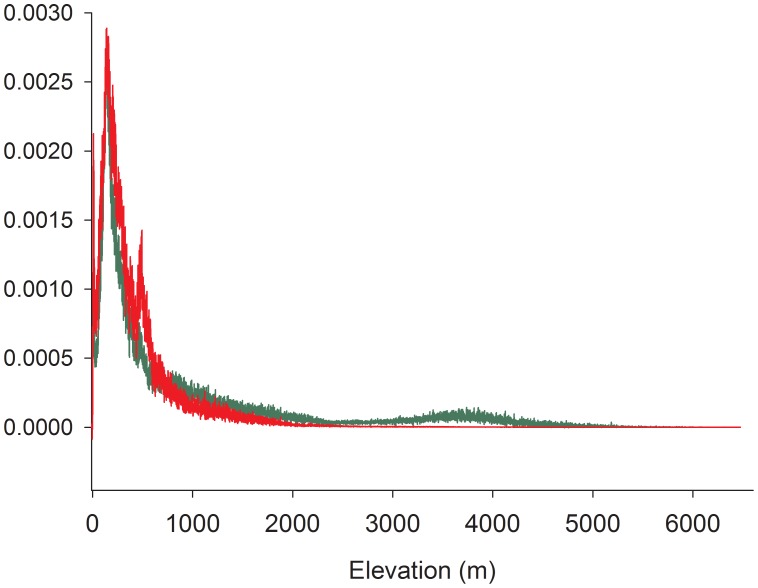
Niche occupancy profile in relation to altitude in central Chile. The red curve is the projected profile from the invaded range (central Chile); the green curve is the projected profile from the native range (California).

The niche occupancy profiles projected from California and central Chile were significantly different to each other (Kolmogorov-Smirnov test; D = 0.53; P<<0.001; Figure 3). The niche occupancy profile projected from California predicted a higher altitudinal range in central Chile (weighted mean elevation: 905 m; Figure 3) compared to the model projected from central Chile (weighted mean elevation: 422 m; Figure 3).

## Discussion

Our results indicate that the climatic niches of *E. californica* in California and central Chile were very similar to each other. This similarity had two particular characteristics: the climatic niche in central Chile was almost completely included within the niche of the native range, and a minor fraction of niche in the invaded range expanded to new environments. However, the larger fraction of unfilled niche predicted from the native niche suggests that *E. californica* hasn't colonized a substantial fraction of the climatic environments in central Chile. An alternative explanation is that the species has colonized only the environments in Chile that overlap between the Californian and Chilean background climates due to the arrival of a biased set of colonizers, with a narrower climatic niche.

Climatic niche conservatism has been largely detected in invasive plant species [7]. If the niches are conserved, then the potential distribution predicted from the native range should be concordant with predictions based on occurrences in the invaded range [51]. However, we can find mismatches between native and invaded range projections due to other ecological factors, even if the climatic niche is conserved (dispersal limitation, biotic interactions) [8], [15]–[19].

In *E. californica*, the projected potential distribution from the native niche differs from the projected distribution from the invaded range. In fact, this area was 2.9 times larger than that projected from the invaded range and extended further north and south (Figure 2). These large differences were not concordant with the slight niche differences between native and invaded range obtained from PCA analysis. In fact, the niche differences were proportionally minors than potential distributions differences between the two ranges. The niche - biotope duality (the conceptual base of the SDMs) [52] explain this asymmetric relationship. The duality means that the niche and the geographic space are closely interconnected [53]. However this duality is not one-to-one [52], [53] i.e. one small and regular zone in the niche space may correspond to many areas in the geographic space [5]. This asymmetric property of the niche- biotope duality has been largely reviewed and discussed in the literature [5], [52], [53], [54].

The causal factor that might explain this discrepancy is that *E. californica* is a “generalist species” spreading over a wide range of habitats from southern Washington to Baja California and from the Channel Islands and Pacific coastline to the Great Basin and regions of the Sonoran Desert [28], thus encompassing a wide range of temperature and precipitation regimes also existent in Chile.

We have estimated that populations of *E. californica* in central Chile, have a positive finite growth rate homogeneously distributed along latitudinal as well as altitudinal gradients [30], suggesting that it performs well across a variety of environments, thus reinforcing the idea that it is a generalist species. If it has no demographic limitations, it could spread covering large geographical ranges as predicted by the native niche model. However, this species could be also dispersal limited based on genetic evidence that indicates that gene flow between populations is limited (Castillo, Master thesis, unpublished data).

High-elevation ecosystems are considered more resistant to plant invasions than other ecosystems [55]; as elevation increases, the environment changes dramatically: mean temperatures decrease, the growing season shortens and UV-B radiation becomes more intense [56]. The differences between the niche occupancy profile (Figure 3) for *E. californica*, from the native and invaded ranges lead us to agree with this assertion. In fact, the mean altitude predicted from California was more than double the central Chile model's, further supporting the non-equilibrium condition of *E. californica* to higher altitudes.

One explanation is that *E. californica* is dispersal limited along elevational gradients (low propagule pressure *sensu* [57]). Another explanation is that before an alien plant species can reach mountain areas, it presumably passes through a “low altitude filter” that excludes those genotypes well-adapted to higher altitudes [58]. In this way, the flow of pre-adapted genes, which evolved at higher altitudes in the native range, is disrupted, and then no expansion would be expected until such adaptations emerge, as it has happened in other species along elevation gradients [55], [59].

From our study, in spite of the climatic niche *E. californica* has been conserved, this species is not in geographical equilibrium and has the potential to continue spreading along Chile, especially into warm regions and to higher altitudes. Reaching geographical equilibrium will depend on its dispersal abilities and to what extent native communities will resist invasion.

## Supporting Information

Table S1 **Occurrence data of** ***Eschscholzia californica*** **in central Chile.**
(PDF)
