# Supplementary material for: Climatic Niche Conservatism and Biogeographical Non-Equilibrium in Eschscholzia californica (Papaveraceae), an Invasive Plant in the Chilean Mediterranean Region
Source: PLoS One. 2014 Aug 19;9(8):e105025. doi: 10.1371/journal.pone.0105025 (PMC4138165; doi:10.1371/journal.pone.0105025)
Supplement: Table S1 — Occurrence data of Eschscholzia californica in central Chile. (PDF) [file pone.0105025.s001.pdf]

Table S1. Occurrence data of *Eschscholzia californica* in central Chile

Species	longitude	latitude
E_californica	-71.228663	-30.610279
E_californica	-71.65623	-33.27027
E_californica	-71.24688	-30.65305
E_californica	-71.13347	-30.60725
E_californica	-71.12877	-30.61432
E_californica	-71.54698	-32.98038
E_californica	-71.6427	-33.3151
E_californica	-71.64835	-33.34067
E_californica	-71.64868	-33.34415
E_californica	-71.66992	-33.37057
E_californica	-71.6896	-33.38595
E_californica	-71.6937	-33.40667
E_californica	-72.79352	-36.07777
E_californica	-72.7901	-36.39245
E_californica	-71.63609	-33.350317
E_californica	-71.56907	-33.03668
E_californica	-72.74453	-36.40952
E_californica	-71.54333	-32.93425
E_californica	-71.54457	-32.95112
E_californica	-71.6375	-33.46895
E_californica	-71.06757	-30.68163
E_californica	-71.53325	-31.58668
E_californica	-71.53718	-32.93392
E_californica	-71.644035	-33.44409
E_californica	-71.62732	-33.47358
E_californica	-71.60212	-33.51212
E_californica	-73.02021	-36.89323
E_californica	-71.51528	-32.92038
E_californica	-71.594175	-33.514749
E_californica	-71.59447	-33.53743
E_californica	-72.70308	-36.46956
E_californica	-71.56817	-33.0434
E_californica	-71.86407	-33.99107
E_californica	-72.66431	-36.42612
E_californica	-71.49395	-32.90928
E_californica	-71.4986	-32.91502
E_californica	-71.56535	-33.53895
E_californica	-71.59223	-33.59755
E_californica	-71.59355	-33.59808
E_californica	-71.595008	-33.626165
E_californica	-71.79434	-33.93949
E_californica	-71.7893	-33.93951
E_californica	-71.78338	-33.94194
E_californica	-71.81463	-33.9589

E_californica	-71.85967	-33.99593
E_californica	-71.85709	-33.99837
E_californica	-72.61649	-35.82924
E_californica	-71.59717	-33.28928
E_californica	-71.5963	-33.6636
E_californica	-71.75807	-33.94229
E_californica	-71.77668	-33.94408
E_californica	-71.74166	-33.94521
E_californica	-71.73802	-33.94561
E_californica	-71.73934	-33.94671
E_californica	-71.73708	-33.9482
E_californica	-71.80165	-33 .94965
E_californica	-71.73432	-33.95227
E_californica	-71.80675	-33.95559
E_californica	-71.4774	-31.87157
E_californica	-71.48143	-31.873
E_californica	-71.49285	-31.87765
E_californica	-71.49293	-31.9027
E_californica	-71.49277	-31.90362
E_californica	-71.49272	-31.90402
E_californica	-71.48068	-32.83603
E_californica	-71.48548	-32.8435
E_californica	-71.55783	-33.054
E_californica	-71.578793	-33.583939
E_californica	-71.73081	-33.96585
E_californica	-71.73097	-33.97198
E_californica	-72.547995	-36.262014
E_californica	-71.46533	-31.86935
E_californica	-71.4683	-31.86975
E_californica	-71.44962	-31.86983
E_californica	-71.45412	-31.87055
E_californica	-71.47357	-31.8707
E_californica	-71.4563	-31.87075
E_californica	-71.48807	-31.87538
E_californica	-71.49163	-31.87665
E_californica	-71.49072	-31.89002
E_californica	-71.49007	-31.9036
E_californica	-71.49852	-31.97958
E_californica	-71.50963	-32.0306
E_californica	-71.51288	-32.04192
E_californica	-71.52118	-32.0666
E_californica	-71.4792	-32.82552
E_californica	-71.47895	-32.82665
E_californica	-71.55787	-33.06008
E_californica	-71.55997	-33.56117
E_californica	-71.5544	-33.56247
E_californica	-71.73039	-33.97775

E_californica	-71.72506	-33.98133
E_californica	-71.72941	-33.98137
E_californica	-71.41698	-31.86888
E_californica	-71.51253	-32.08962
E_californica	-71.50982	-32.09567
E_californica	-71.49992	-32.13632
E_californica	-71.51498	-32.14516
E_californica	-71.50948	-32.22943
E_californica	-71.5012	-32.23465
E_californica	-71.48037	-32.81867
E_californica	-71.5627	-33.13555
E_californica	-71.71671	-70 .00032
E_californica	-71.72452	-34.01291
E_californica	-71.7279	-34.01668
E_californica	-71.98827	-34.38581
E_californica	-71.990475	-34.386193
E_californica	-71.99633	-34.38666
E_californica	-73.051713	-36.853593
E_californica	-71.39205	-31.86415
E_californica	-71.4142	-31.86953
E_californica	-71.41278	-31.86987
E_californica	-71.41005	-31.87058
E_californica	-71.40422	-31.87145
E_californica	-71.40555	-31.87185
E_californica	-71.48877	-31.96472
E_californica	-71.49615	-32.23802
E_californica	-71.48323	-32.78365
E_californica	-71.47888	-32.79194
E_californica	-71.47635	-32.81242
E_californica	-71.50802	-33.57707
E_californica	-71.49965	-33.58362
E_californica	-71.49305	-33.59153
E_californica	-71.48953	-33.59768
E_californica	-73.03022	-36.91397
E_californica	-70 .88013	-30.71033
E_californica	-71.3884	-31.86132
E_californica	-71.3962	-31.86687
E_californica	-71.3979	-31.86848
E_californica	-71.46677	-32.5292
E_californica	-71.46757	-32.7406
E_californica	-71.47335	-32.74385
E_californica	-71.47447	-32.80122
E_californica	-71.5931	-33.21363
E_californica	-71.48048	-33.60205
E_californica	-71.4601	-33.61065
E_californica	-71.7379	-34.10535
E_californica	-71.73911	-34.10835

E_californica	-71.7367	-34.11275
E_californica	-71.73384	-34.11392
E_californica	-71.72739	-34.1291
E_californica	-71.72793	-34.13223
E_californica	-71.94413	-34.3574
E_californica	-71.46285	-32.28778
E_californica	-71.4455	-32.34302
E_californica	-71.44227	-32.34658
E_californica	-71.44538	-32.34727
E_californica	-71.44177	-32.34778
E_californica	-71.44437	-32.34788
E_californica	-71.4417	-32.3479
E_californica	-71.44217	-32.34917
E_californica	-71.4565	-32.73498
E_californica	-71.46442	-32.739
E_californica	-71.4487	-33.6116
E_californica	-71.4302	-33.61868
E_californica	-71.4256	-33.62028
E_californica	-71.72885	-34.13634
E_californica	-71.73173	-34.14625
E_californica	-72.43532	-35.388963
E_californica	-71.27727	-31.66983
E_californica	-71.27818	-31.67
E_californica	-71.2838	-31.6718
E_californica	-71.45795	-32.3206
E_californica	-71.45233	-32.33612
E_californica	-71.45098	-32.33812
E_californica	-71.47355	-32.52247
E_californica	-71.46208	-32.54565
E_californica	-71.44263	-32.5787
E_californica	-71.44865	-32.73092
E_californica	-71.44269	-32.73272
E_californica	-71.41882	-33.6201
E_californica	-71.73148	-34.17222
E_californica	-71 .73349	-34.18488
E_californica	-71.66415	-34.2057
E_californica	-71.70316	-34.20577
E_californica	-71.72663	-34.21153
E_californica	-71.80052	-34.29789
E_californica	-72.02005	-34.544566
E_californica	-72.9629	-37.05249
E_californica	-72.95642	-37.08586
E_californica	-71.23567	-31.65947
E_californica	-71.2389	-31.6602
E_californica	-71.26205	-31.66712
E_californica	-71.28563	-31.67665
E_californica	-71.27927	-31.70292

E_californica	-71.42917	-32.35205
E_californica	-71.40998	-32.35898
E_californica	-71.4211	-32.51107
E_californica	-71.45507	-32.5579
E_californica	-71.42348	-32.6348
E_californica	-71.42135	-32.72555
E_californica	-71.4308	-32.72817
E_californica	-71.3941	-33.63082
E_californica	-71.62688	-34.24671
E_californica	-71.7307	-34.26195
E_californica	-71.84966	-34.30777
E_californica	-71.85898	-34.31343
E_californica	-71.86246	-34.31569
E_californica	-71.38388	-32.38377
E_californica	-71.3829	-32.38397
E_californica	-71.46493	-32.54037
E_californica	-71.44832	-32.57462
E_californica	-71.42805	-32.6192
E_californica	-71.39291	-33.38541
E_californica	-71.3973	-33.41572
E_californica	-71.3565	-33.65023
E_californica	-71.3369	-33.65372
E_californica	-71.61794	-34.26887
E_californica	-71.62227	-34.29788
E_californica	-71.86699	-34.32001
E_californica	-71.87516	-34.33799
E_californica	-71.87095	-34.34918
E_californica	-71.86864	-34.35682
E_californica	-72.1816	-34.91298
E_californica	-72.34873	-35.94674
E_californica	-71.19043	-31.64675
E_californica	-71.19658	-31.6488
E_californica	-71.20418	-31.65132
E_californica	-71.20594	-31.65167
E_californica	-71.20607	-31.65195
E_californica	-71.20738	-31.65238
E_californica	-71.20857	-31.65278
E_californica	-71.21297	-31.6542
E_californica	-71.21712	-31.65557
E_californica	-71.21797	-31.65585
E_californica	-71.22158	-31.65632
E_californica	-71.23068	-31.65717
E_californica	-71.27437	-31.7109
E_californica	-71.27562	-31.72147
E_californica	-71.27498	-31.72215
E_californica	-71.37392	-32.39403
E_californica	-71.40712	-32.69357

E_californica	-71.40602	-32.7013
E_californica	-71.61784	-34.32107
E_californica	-71.61283	-34.33788
E_californica	-71.61353	-34.34817
E_californica	-72.04469	-34.77791
E_californica	-72.18156	-34.97684
E_californica	-72.32198	-35.96218
E_californica	-72.4307	-36.65426
E_californica	-71.18562	-31.640076
E_californica	-71.17595	-31.64253
E_californica	-71.17707	-31.64322
E_californica	-71.1833	-31.64435
E_californica	-71.401	-32.49798
E_californica	-71.404457	-32.499259
E_californica	-71.45747	-32.5478
E_californica	-71.45535	-32.54893
E_californica	-71.45384	-34.2962
E_californica	-71.48259	-34.29946
E_californica	-71.61437	-34.32706
E_californica	-72.290296	-35.977775
E_californica	-71.14563	-31.62088
E_californica	-71.3117	-31.74848
E_californica	-71.31152	-31.74883
E_californica	-71.36228	-32.40483
E_californica	-71.32785	-32.42997
E_californica	-71.32722	-32.43138
E_californica	-71.3211	-32.44477
E_californica	-71.32488	-32.44832
E_californica	-71.35975	-32.46457
E_californica	-71.3632	-32.46705
E_californica	-71.37547	-32.481
E_californica	-71.38057	-32.48682
E_californica	-71.31345	-33.44933
E_californica	-72.27817	-35.95739
E_californica	-72.27338	-35.95807
E_californica	-72.25936	-35.96033
E_californica	-71.12477	-31.5968
E_californica	-71.12523	-31.59777
E_californica	-71.1251	-31.59798
E_californica	-71.12887	-31.60973
E_californica	-71.1324	-31.61373
E_californica	-71.13728	-31.61662
E_californica	-71.13989	-31.61783
E_californica	-71.14518	-31.63948
E_californica	-71.1665	-31.74782
E_californica	-71.16492	-31.75105
E_californica	-71.164	-31.75223

E_californica	-71.16323	-31 .75292
E_californica	-71.15893	-31.75703
E_californica	-71.30215	-32.4486
E_californica	-71.33115	-32.45347
E_californica	-71.37313	-33.43328
E_californica	-71.34068	-34.28517
E_californica	-71.36785	-34.28877
E_californica	-72.00499	-34.86523
E_californica	-71.11068	-31.57643
E_californica	-71.11502	-31.58328
E_californica	-71.12768	-31.60788
E_californica	-71.1574	-31.7633
E_californica	-71.1573	-31.76388
E_californica	-71.27513	-32.44918
E_californica	-71.29105	-32.45015
E_californica	-72.71989	-37.27316
E_californica	-71.26552	-32.44638
E_californica	-71.322498	-32.582713
E_californica	-72.27023	-36.71436
E_californica	-72.33485	-36.88941
E_californica	-72.64627	-37.36143
E_californica	-71.15873	-31.80792
E_californica	-71.16083	-31.81412
E_californica	-71.26973	-32.4506
E_californica	-72.34661	-36.93108
E_californica	-72.60088	-37.427
E_californica	-71.16338	-31.8191
E_californica	-71.20863	-32.31585
E_californica	-71.25597	-32.45407
E_californica	-71.25607	-32.45573
E_californica	-71.25678	-32.46577
E_californica	-71.25705	-32.46953
E_californica	-71.25712	-32.47048
E_californica	-71.25765	-32.478
E_californica	-71.25863	-32.49252
E_californica	-71.25882	-32.49473
E_californica	-71.25893	-32.49648
E_californica	-71.25898	-32.49712
E_californica	-71.25955	-32.50538
E_californica	-71.25997	-32.51112
E_californica	-70.98083	-34.45699
E_californica	-70.95128	-34.54373
E_californica	-70.96274	-34.55357
E_californica	-70.96416	-34.55604
E_californica	-70.97126	-34.5737
E_californica	-70.96605	-34.58711
E_californica	-72.605631	-37.698233

E_californica	-71.26538	-31 .53732
E_californica	-71.263361	-32.539381
E_californica	-71.26592	-32.53953
E_californica	-71.26708	-32.55303
E_californica	-71.2868	-32.93888
E_californica	-71.28592	-32.94322
E_californica	-71.28348	-32.97108
E_californica	-70.97677	-34.3689
E_californica	-70.95505	-34.45492
E_californica	-70.944677	-34.537435
E_californica	-70.98897	-34.62477
E_californica	-72.39092	-37.16713
E_californica	-72.39242	-37.18582
E_californica	-72.38912	-37.20425
E_californica	-72.33647	-38.23946
E_californica	-71.08626	-31.29926
E_californica	-71.04868	-31.77077
E_californica	-71.26617	-32.55477
E_californica	-71.26378	-32.55922
E_californica	-71.26195	-32.56265
E_californica	-71.26017	-32.566
E_californica	-71.28878	-32.92857
E_californica	-71.28875	-32.93
E_californica	-71.2849	-32.95295
E_californica	-71.28682	-32.95492
E_californica	-71.2862	-32.9597
E_californica	-71.28508	-32.96157
E_californica	-71.24688	-33.0057
E_californica	-71.24863	-33.00587
E_californica	-70.94577	-34.34845
E_californica	-70.92438	-34.41498
E_californica	-70.91562	-34.4963
E_californica	-71.08484	-31.30067
E_californica	-71.08504	-31.30108
E_californica	-71.08514	-31.30128
E_californica	-71.08421	-31.30161
E_californica	-71.08372	-31.30162
E_californica	-71.08357	-31.30405
E_californica	-71.08351	-31.30451
E_californica	-71.0796	-31.30547
E_californica	-71.07926	-31.30564
E_californica	-71.0367	-31.7765
E_californica	-71.03492	-31.77663
E_californica	-71.03575	-31.77683
E_californica	-71.03358	-31.77707
E_californica	-71.01028	-31 .7771
E_californica	-71.01987	-31.77722

E_californica	-71.03285	-32 .77742
E_californica	-71.03017	-31.7785
E_californica	-71.02845	-31.77898
E_californica	-71.1354	-31.929
E_californica	-71.1365	-31.9327
E_californica	-71.1346	-31.93552
E_californica	-71.13522	-31.94968
E_californica	-71.27795	-32.9205
E_californica	-71.28095	-32.92398
E_californica	-71.28227	-32.97407
E_californica	-70.9075	-34.40268
E_californica	-70.87577	-34.40765
E_californica	-70.87748	-34.41062
E_californica	-70.88031	-34.41541
E_californica	-70.88582	-34.42829
E_californica	-70.88736	-34.43323
E_californica	-70.99402	-34.66298
E_californica	-71.0012	-34.6725
E_californica	-71.1324	-31.93892
E_californica	-71.1352	-31.95135
E_californica	-71.13733	-31.95508
E_californica	-71.25462	-32.5781
E_californica	-71.25285	-32.57955
E_californica	-71.25053	-32.58157
E_californica	-70.85828	-34.37711
E_californica	-70.85919	-34.37871
E_californica	-70.86003	-34.3802
E_californica	-70.86963	-34.39696
E_californica	-72.30295	-37.78655
E_californica	-71.26303	-32.90683
E_californica	-71.2078	-33.01368
E_californica	-71.21443	-33.0156
E_californica	-71.131985	-33.932495
E_californica	-70.86455	-34.34039
E_californica	-70.84985	-34.34442
E_californica	-70.84064	-34.34642
E_californica	-70.84226	-34.34917
E_californica	-70.84383	-34.3519
E_californica	-70.84482	-34.35372
E_californica	-70.84694	-34.35739
E_californica	-70.84942	-34.36163
E_californica	-72.27427	-37.65224
E_californica	-70.99473	-31.77488
E_californica	-71.14073	-31.97408
E_californica	-71.14175	-31.97578
E_californica	-71.1683	-32 .07217
E_californica	-71.1695	-32.07245

E_californica	-71.16892	-32 .07252
E_californica	-71.16863	-32.07252
E_californica	-71.1695	-32.07253
E_californica	-71.16912	-32.07255
E_californica	-71.16962	-32.07258
E_californica	-71.1694	-32.07277
E_californica	-71.16827	-32.07282
E_californica	-71.16958	-32.0729
E_californica	-71.24958	-32.58348
E_californica	-71.24113	-32.90027
E_californica	-70.82507	-34.30434
E_californica	-70.82637	-34.3104
E_californica	-70.83218	-34.328
E_californica	-70.83323	-34.33104
E_californica	-70.83471	-34.33529
E_californica	-70.83657	-34.3393
E_californica	-71.03654	-34.72836
E_californica	-71.22913	-32.6448
E_californica	-71.2266	-32.64835
E_californica	-71.23285	-32.89227
E_californica	-71.23655	-32.8965
E_californica	-71.17362	-33.01927
E_californica	-71.16937	-33.02093
E_californica	-71.16595	-33.0223
E_californica	-70.81535	-34.27676
E_californica	-70.81674	-34.27922
E_californica	-70.81985	-34.28464
E_californica	-70.82192	-34.28805
E_californica	-70.82278	-34.29267
E_californica	-70.82349	-34.29639
E_californica	-71.04069	-34.74583
E_californica	-71.04072	-34.74849
E_californica	-71.0412	-34.75213
E_californica	-71.92702	-36.39258
E_californica	-71.23227	-32.6343
E_californica	-71.23207	-32.63595
E_californica	-71.21848	-32.6604
E_californica	-71.21468	-32.66743
E_californica	-71.21335	-32.67088
E_californica	-71.2126	-32.67397
E_californica	-71.2113	-32.67955
E_californica	-71.22115	-32.80965
E_californica	-71.22202	-32.81088
E_californica	-71.22242	-32.8273
E_californica	-71.22207	-32.82793
E_californica	-71.22093	-32.83003
E_californica	-71.22043	-32.83095

E_californica	-71.21995	-32.833
E_californica	-71.22423	-32.84603
E_californica	-71.2243	-32.84933
E_californica	-71.2317	-32.8835
E_californica	-71.232	-32.88582
E_californica	-71.2324	-32.88922
E_californica	-71.15202	-33.02602
E_californica	-71.14967	-33.02673
E_californica	-71.14773	-33.02737
E_californica	-71.14652	-33.02777
E_californica	-71.14362	-33.02882
E_californica	-71.14192	-33.02955
E_californica	-70.8426	-34.18711
E_californica	-70.80127	-34.25215
E_californica	-70.8034	-34.25588
E_californica	-70.81	-34.26714
E_californica	-70.81243	-34.27168
E_californica	-71.04202	-34.75853
E_californica	-71.89736	-38.43634
E_californica	-71.23983	-32.59302
E_californica	-71.23678	-32.59318
E_californica	-71.23518	-32.59332
E_californica	-71.23453	-32.59398
E_californica	-71.2358	-32.59415
E_californica	-71.20567	-32.70782
E_californica	-71.2015	-32.72967
E_californica	-71.19715	-32.74602
E_californica	-71.19632	-32.74995
E_californica	-71.20622	-32.79973
E_californica	-71.20968	-32.80077
E_californica	-71.21617	-32.8035
E_californica	-71.2236	-32.81698
E_californica	-71.22277	-32.82098
E_californica	-71.14005	-33.0305
E_californica	-71.13888	-33.03155
E_californica	-71.13753	-33.0325
E_californica	-70.946008	-33.672135
E_californica	-70.93826	-33.68169
E_californica	-70.79182	-34.23519
E_californica	-70 .7977	-34.2455
E_californica	-70.79996	-34.24989
E_californica	-72.02696	-37.66335
E_californica	-72.03191	-37.6673
E_californica	-71.06888	-32.35291
E_californica	-71.23272	-32.61792
E_californica	-71.23118	-32.62445
E_californica	-71.195	-32.7571

E_californica	-71.17715	-32.7807
E_californica	-71.17578	-32.78182
E_californica	-71.19613	-32.79538
E_californica	-71.19932	-32.79688
E_californica	-71.13412	-33.03397
E_californica	-71.13283	-33.0347
E_californica	-71.12967	-33.03652
E_californica	-71.12832	-33.03728
E_californica	-71.12462	-33.03947
E_californica	-71.12218	-33.04235
E_californica	-71.11982	-33.04477
E_californica	-71.11423	-33.05012
E_californica	-71.113	-33.05093
E_californica	-71.11197	-33.05162
E_californica	-71.1115	-33.05192
E_californica	-70.78384	-34.13235
E_californica	-70.78459	-34.13545
E_californica	-70.78533	-34.13859
E_californica	-70.78824	-34.14765
E_californica	-70.79113	-34.15432
E_californica	-70.79374	-34.16182
E_californica	-70.79266	-34.17189
E_californica	-70.79162	-34.17476
E_californica	-70.78799	-34.18771
E_californica	-70.78492	-34.2037
E_californica	-70.78901	-34.21824
E_californica	-70.79012	-34.21993
E_californica	-70.79063	-34.22155
E_californica	-71.06172	-34.81578
E_californica	-71.23813	-32.60985
E_californica	-71.23598	-32.61077
E_californica	-71.17355	-32.78368
E_californica	-71.17198	-32.78495
E_californica	-71.16792	-32.78772
E_californica	-71.17845	-32.79202
E_californica	-70.951701	-33.540134
E_californica	-70.77022	-34.08125
E_californica	-70.771	-34.08176
E_californica	-70.77939	-34.1008
E_californica	-70.78027	-34.10932
E_californica	-70.78009	-34.11176
E_californica	-71 .06636	-34.82045
E_californica	-71.06825	-34.82234
E_californica	-71.07442	-34.82568
E_californica	-71.78935	-36.08538
E_californica	-71.10133	-33.05068
E_californica	-71.10068	-33.05087

E_californica	-71.10273	-33.05137
E_californica	-71.09478	-33.05318
E_californica	-71.09198	-33.05412
E_californica	-70 .72957	-34.13595
E_californica	-71.23877	-32.60012
E_californica	-71.23968	-32.60097
E_californica	-71.09063	-33.05457
E_californica	-71.08578	-33.0571
E_californica	-70.7335	-34.0482
E_californica	-70.74371	-34.05288
E_californica	-71.71746	-35.99838
E_californica	-71.08198	-33.05867
E_californica	-71.07738	-33.06112
E_californica	-71.07535	-33.06193
E_californica	-70.75187	-33.83863
E_californica	-70.74763	-33.8591
E_californica	-70.73549	-33.88881
E_californica	-70.73325	-33.90925
E_californica	-70.71297	-33.95003
E_californica	-70.70874	-33.96616
E_californica	-70.70724	-33.97178
E_californica	-70.70297	-34.01133
E_californica	-71.15904	-34.90363
E_californica	-71.66547	-35.93699
E_californica	-71.06823	-33.0628
E_californica	-71.07398	-33.06282
E_californica	-71.06997	-33.06327
E_californica	-71.07288	-33.06343
E_californica	-71.07117	-33.0635
E_californica	-70.74535	-33.7847
E_californica	-70.64693	-34.19326
E_californica	-71.69147	-35.53763
E_californica	-71.6937667	-35.54275
E_californica	-71.69515	-35.55845
E_californica	-71.69694	-35.56501
E_californica	-71.62786	-35.84007
E_californica	-71.62965	-35.84071
E_californica	-70.3171389	-33.3523056
E_californica	-70.31725	-33.3526944
E_californica	-70.3169444	-33.3528889
E_californica	-70.3169444	-33.3531111
E_californica	-70.318902	-33.355964
E_californica	-70.288692	-33.610387
E_californica	-71.59045	-35.85032
E_californica	-71.49412	-35.2718
E_californica	-71.55708	-35.33428
E_californica	-71.56004	-35.33662

E_californica	-71.589813	-35.369764
E_californica	-71.59358	-35.37185
E_californica	-71.60467	-35.3835
E_californica	-71 .61834	-35.39776
E_californica	-71.64177	-35.44406
E_californica	-71.61894	-35.58616
E_californica	-71.60738	-35.59088
E_californica	-71.54204	-35.83405
E_californica	-71.36702	-38.45692
E_californica	-71.06577	-33.06358
E_californica	-71.06565	-33.06505
E_californica	-70.902108	-33.523966
E_californica	-71.2105	-34.98008
E_californica	-71.22991	-34.99308
E_californica	-71.23062	-34.99341
E_californica	-71.24437	-35.00909
E_californica	-71.24658	-35.01178
E_californica	-71.27906	-35.04903
E_californica	-71.28507	-35.05223
E_californica	-71.3131	-35.08724
E_californica	-71.33681	-35.11694
E_californica	-71.37213	-35.14817
E_californica	-71.37744	-35.15531
E_californica	-71.38377	-35.16517
E_californica	-71.38568	-35.17202
E_californica	-71.39029	-35.1833
E_californica	-71.39596	-35.1929
E_californica	-71.41494	-35.2141
E_californica	-71.42161	-35.21919
E_californica	-71.48655	-35.26196
E_californica	-71.50271	-35.28458
E_californica	-71.5069	-35.29025
E_californica	-71.51673	-35.30223
E_californica	-71.05282	-33.05972
E_californica	-71.05208	-33.06035
E_californica	-70.72607	-33.68086
E_californica	-71.22979	-35.00097
E_californica	-71.2502	-35.02935
E_californica	-71.2512	-35.03072
E_californica	-71.25232	-35.03236
E_californica	-71.46987	-35.82087
E_californica	-71.04228	-33.05747
E_californica	-71.04317	-33.05785
E_californica	-70.71388	-33.65289
E_californica	-70.71805	-33.66218
E_californica	-71.04677	-33.05868
E_californica	-71.04957	-33.05888

E_californica	-71.04838	-33.05973
E_californica	-71.0199	-33.0672
E_californica	-70.91978	-33.13348
E_californica	-70.91808	-33.13725
E_californica	-70.91757	-33.13827
E_californica	-70.91573	-33.14203
E_californica	-70.9131	-33.14568
E_californica	-70.90892	-33.15075
E_californica	-70.90647	-33.15147
E_californica	-70.90295	-33.15337
E_californica	-70.90088	-33.15533
E_californica	-70.89625	-33.15577
E_californica	-70.90005	-33.15618
E_californica	-70.89818	-33.15818
E_californica	-70.89678	-33.15967
E_californica	-70.89557	-33.16095
E_californica	-70.89358	-33.1631
E_californica	-70.88947	-33.1674
E_californica	-70.8888	-33.16743
E_californica	-70.831507	-33.245655
E_californica	-70.70363	-33.55199
E_californica	-70.71145	-33.6471
E_californica	-70.71254	-33.64998
E_californica	-70.56386	-34.19524
E_californica	-71.37318	-35.7005
E_californica	-71.30412	-35.70915
E_californica	-71.03278	-33.05947
E_californica	-71.03148	-33.05947
E_californica	-71.03027	-33.05952
E_californica	-71.02838	-33.06135
E_californica	-71.0311	-33.0634
E_californica	-71.02802	-33.06348
E_californica	-70.92938	-33.09625
E_californica	-70.92957	-33.09842
E_californica	-70.92602	-33.1177
E_californica	-70.9247	-33.12217
E_californica	-70.92315	-33.126
E_californica	-70.92117	-33.13043
E_californica	-70.8867	-33.16397
E_californica	-70.89118	-33.16572
E_californica	-70.69116	-33.54433
E_californica	-70.57117	-34.19618
E_californica	-71.24429	-35.72791
E_californica	-70.93858	-33.07525
E_californica	-70.93002	-33.07562
E_californica	-70.92963	-33.08797
E_californica	-70.92895	-33.09135

E_californica	-70.87005	-33.14623
E_californica	-70.87397	-33.14925
E_californica	-70.75948	-33.23385
E_californica	-70.7464	-33.26308
E_californica	-70.73528	-33.28782
E_californica	-70.3253611	-33.3495278
E_californica	-70.3252222	-33.3495278
E_californica	-70.98808	-33.06542
E_californica	-70.98423	-33.06843
E_californica	-70.99557	-33.06908
E_californica	-70.98028	-33.06975
E_californica	-70.97678	-33.07035
E_californica	-70.97853	-33.07048
E_californica	-70.97142	-33.07232
E_californica	-70.95823	-33.0756
E_californica	-70.95612	-33.07633
E_californica	-70.95185	-33.077
E_californica	-70.94777	-33.07742
E_californica	-70.84968	-33.13108
E_californica	-70.75588	-33.24175
E_californica	-70.74957	-33.25597
E_californica	-70.336489	-33.342216
E_californica	-70.629631	-33.415759
E_californica	-70.644651	-33.419738
E_californica	-70.304364	-33.623322
E_californica	-70.301546	-32.907795
E_californica	-70.96055	-33.0748
E_californica	-70.82323	-33.1198
E_californica	-70.82607	-33.11993
E_californica	-70.82177	-33.12295
E_californica	-70.82042	-33.12448
E_californica	-70.81578	-33.12982
E_californica	-70.8148	-33.13092
E_californica	-70.620288	-33.407607
E_californica	-70.631114	-33.646168
E_californica	-70.80025	-33.14738
E_californica	-70.79743	-33.15055
E_californica	-70.3383056	-33.3388889
E_californica	-70.58805	-33.64232
E_californica	-70.585877	-33.655912
E_californica	-70.58436	-33.67442
E_californica	-70.579521	-33.682019
E_californica	-70.57894	-33.68276
E_californica	-70.5785	-33.68456
E_californica	-70.57561	-33.68788
E_californica	-70.57371	-33.69025
E_californica	-70.56425	-33.70654

E_californica	-70.55005	-33.70691
E_californica	-70.5518	-33.70821
E_californica	-70.55988	-33.70848
E_californica	-70.55799	-33.70933
E_californica	-70.55439	-33.71104
E_californica	-70.55285	-33.71171
E_californica	-70.58085	-33.48804
E_californica	-70.58001	-33.48806
E_californica	-70.54444	-33.70795
E_californica	-70.79133	-33.17387
E_californica	-70.354786	-33.348567
E_californica	-70.57414	-33.60218
E_californica	-70.496602	-33.597714
E_californica	-70.166727	-33.816601
E_californica	-70.47784	-33.586967
E_californica	-70.339716	-33.678771
E_californica	-70.272628	-33.76736
E_californica	-70.191993	-33.814655
E_californica	-70.360333	-33.3445
E_californica	-70.362858	-33.346358
E_californica	-70.378298	-33.359499
E_californica	-70.519887	-33.481473
E_californica	-70.408939	-33.588142
E_californica	-70.349664	-33.677766
E_californica	-70.333216	-33.707977
E_californica	-70.408864	-33.370718
E_californica	-70.646941	-33.145748
E_californica	-70.458083	-33.370849
E_californica	-70.7324833	-32.6905833
